# Grisolle

**Published:** 1869-04-01

**Authors:** 


### OBITUARY.

GRISOLLE.—After three years of suffering, in consequence of an attack of apoplexy, this distinguished physician died on the 10th of February, in the fifty-eighth year of his age. He was borne to his last resting-place by his fellow-members of the Faculty of Medicine, Paris.

